# Tracheal Squamous Cell Carcinoma Treated with Tracheal Resection and Anastomosis in a Cat

**DOI:** 10.1155/2020/8818660

**Published:** 2020-08-07

**Authors:** Zachary A. Miller, Sheldon Padgett, Alex Terreros, Emily Pearce

**Affiliations:** Metropolitan Veterinary Hospital, Akron, OH, USA

## Abstract

A 10-year-old male castrated domestic shorthair cat presented for a suspected tracheal mass. Radiographs confirmed an intraluminal tracheal mass. Tracheal resection and anastomosis of 5 tracheal rings was performed with minimal, mild intraoperative complications and no postoperative complications. Histopathology of the tracheal mass revealed a diagnosis of squamous cell carcinoma (SCC) with incomplete margins both cranially and caudally. Further treatment, including surgical revision, radiation therapy, or chemotherapy, was recommended. At the time of publication, no further treatment has been initiated, and a scheduled consultation with the oncologist has been canceled. The cat is doing well at home with no reported signs of recurrence 120 days postoperatively. This is the first report of a cat with a tracheal SCC to be treated with a tracheal resection and anastomosis and only the third feline tracheal SCC to be treated in the veterinary literature.

## 1. Introduction

Feline tracheal masses are a rare but serious life-threatening condition. In the English veterinary literature, there have been only 55 reported cases in 31 separate publications. Fourteen of the fifty-five cases received no treatment and were either euthanized or died within a week of presentation [1–11]. Feline tracheal inflammatory masses are extremely rare in the cat with only 4 reported including a lymphoid hyperplasia [1], a lymphoplasmacytic inflammatory polyp [1], a mycobacterial granuloma [12], and an inflammatory polyp [13]. The remaining 51 cases have been diagnosed as neoplastic. The most common feline intraluminal tracheal neoplasms include lymphoma (18) [1, 2, 4, 14–22], adenocarcinoma (15) [1, 3, 4, 8, 21, 23–28], and squamous cell carcinoma (6) [1, 4–8]. The rest include various carcinomas including basal cell carcinomas (2) [4, 29], seromucinous carcinoma (1) [9], neuroendocrine carcinoma (1) [10], and unclassified carcinomas (4) [4, 8, 9, 30], as well as one each of leiomyosarcoma [31], histiocytic sarcoma (disseminated) [11], and a plasmacytoma [8]. It has been suggested that the preferred method of treatment for most feline tracheal masses, aside from lymphoma, is to perform a tracheal resection and anastomosis (R&A), should the anatomy allow [4, 8, 9, 29, 31]. Of the 10 previous cats treated by tracheal R&A due to a tracheal mass, 8 recovered well with clinical remission for a variable amount of time [4, 8, 18–21, 25, 29, 31]. Despite this information and despite R&A being labeled as the gold standard for most tracheal masses, there have been no reported cases of feline tracheal squamous cell carcinomas (SCC) treated with tracheal R&A in the veterinary literature.

Squamous cell carcinoma is the third most common diagnosis for feline tracheal masses, accounting for 11.1% of the previously reported feline tracheal masses. Of the reported 6 cases, 4 were euthanized with no treatment due to the poor prognosis [1, 5–7]. The remaining two both had endoscopic debulking procedures performed [4, 8]. Both recovered well and were discharged with normal respiratory function; however respiratory signs recurred at 4 and 8 weeks after the initial procedure due to local recurrence. One cat was euthanized at 4 weeks [8], while the other had a second endoscopic debulking procedure performed 60 days after the initial procedure [4]. Clinical signs reoccurred 20 days later, and the cat was euthanized shortly after [4]. This current report describes the first tracheal R&A performed for a feline tracheal SCC, the immediate postoperative outcome, and shows an increased disease-free interval than achieved when compared to the debulking procedures noted above.

## 2. Case Presentation

A 10-year-old male castrated domestic shorthair cat presented to the author's hospital for progressive wheezing, increased respiratory effort, and dyspnea of 3 month's duration. On the day of presentation, the owners noted the cat occasionally had open mouth breathing and was breathing harder after walking up/down the stairs. The cat was taken to the primary care veterinarian 2 months prior, where radiographs revealed a soft tissue opacity in the trachea at the thoracic inlet. Tracheal collapse was suspected, and the cat was prescribed doxycycline (5 mg/kg PO q12h) and theophylline (100 mg PO q24h) which had no immediate effect on the clinical signs. The cat had an adverse reaction to the medications, causing hypersalivation, vocalization, and hyperactivity causing the owners to discontinue the medications. Following this, the primary care veterinarian recommended a surgical consultation.

On initial presentation, the cat was bright, alert, and responsive with pink and moist mucous membranes and a body condition score of 4/9. There was increased inspiratory and expiratory effort throughout the full respiratory cycle with wet sounding respiratory noises consistent with a partial tracheal obstruction. Cardio-thoracic auscultation revealed only moderate referred tracheal noises. The rest of the physical examination was unremarkable. The primary care veterinarian's radiographs were reviewed and were found to be most consistent with a soft tissue partial obstruction of the trachea at the thoracic inlet. Unfortunately, the thoracic limbs were positioned over the area of interest, making the radiographs difficult to fully assess.

Recommendations included a computed tomography of the cervical and thoracic region for surgical planning as well as tracheoscopy to obtain a small cup biopsy for histopathologic diagnosis with the possibility for debulking under the same anesthesia. Based on the result of the histopathologic diagnosis, either chemotherapy (for lymphoma) or surgical tracheal R&A would be recommended. Alternatively, a tracheal R&A with histology of the mass could be performed without first obtaining a definitive diagnosis. Additionally, tracheal stenting was offered as a palliative maneuver. After discussing the risks versus benefits of different options, the owners decided to proceed with repeat radiographs and a tracheal R&A, forgoing any preliminary advanced diagnostics.

**Figure 1:**
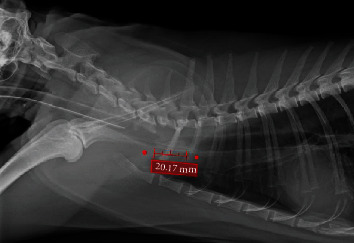
Preoperative lateral thoracic radiographs of a 10 yo MC domestic shorthair cat with a suspected intraluminal tracheal mass. The mass effect measures at 2 cm and appears to be broad based arising from the ventral wall of the trachea.

The cat was hospitalized and placed in an oxygen cage overnight in preparation for surgery the following day. A complete blood count and a chemistry profile showed a mild metabolic alkalosis with a total CO2 of 26 mmol/L (likely compensation to chronic hypoventilation and hypercapnia), a mildly elevated total protein (9.1), and a mild leukocytosis (27.1 k) due to a moderate eosinophilia (6 k), mild monocytosis (800), and mild neutrophilia (16.5 k). The remainder of the biochemistry panel was unremarkable. Vitals were normal (T: 100.5 F, P: 150 bpm, R: 24 bpm), and the cat's breathing pattern remained static overnight. The next morning, an IV catheter was placed, and the cat was premedicated with a bolus of fentanyl (4 mcg/kg IV once), induced with Valium (0.25 mg/kg IV) and propofol (4 mg/kg IV to effect). The patient was intubated routinely, using a 5.0 mm endotracheal tube, terminating rostral to the tracheal mass. Anesthesia was maintained with Isoflurane and a fentanyl continuous rate infusion (4-8 mcg/kg/h IV). When the trachea was not intubated during the procedure (see below), propofol was used IV for total intravenous anesthesia (TIVA) as needed to maintain an adequate plane of anesthesia until reintubation. The cat was placed on a mechanical ventilator with the pressure set to 5 cmH2O and a rate of 5 breaths per minute. The cat was given prophylactic antibiotics of cefazolin (22 mg/kg IV q90 minutes) twice during the surgery. Intravenous isotonic crystalloids were run at 5 ml/kg/h during the surgery. Monitoring and support included capnography, pulse oximetry, esophageal temperature, blood pressure (doppler and oscillometric), and telemetry as well as a forced warm air circulator and a fluid line warmer for thermal support. Preoperative radiographs were obtained (Figure 1) which showed an approximately 2 cm intraluminal tracheal mass at the level of the thoracic inlet obstructing >50% of the tracheal lumen.

**Figure 2:**
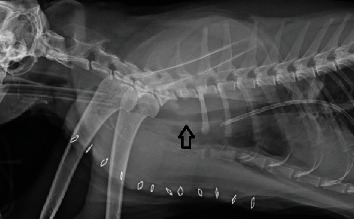
Postoperative lateral thoracic radiographs of a 10 yo MC domestic shorthair cat after a tracheal resection and anastomosis to remove a tracheal mass. The arrow is pointing to the anastomotic site that has slight intraluminal narrowing.

A ventral, cervical, midline incision with a partial cranial sternotomy was performed. The cranial half-sternotomy was made using a battery-operated oscillating saw. The trachea was freed from surrounding tissues using blunt dissection. Upon inspection of the trachea, there was a 2 cm lymph node on the left lateral aspect of the trachea just cranial to the thoracic inlet. At the thoracic inlet, there was a firm, multinodular mass associated with approximately 2.2 cm of the trachea that was obstructing greater than 50% of the lumen. The local cervical lymph node was removed and submitted for histopathology. After placing vessel loops, stay sutures, and preplaced appositional sutures, the trachea was transected caudal to the mass. A sterile 4.5 mm tube was then placed through the surgical site and into the caudal tracheal segment to allow for continued mechanical ventilation and maintenance with isoflurane and oxygen. Approximately, 3 cm (corresponding to 5 tracheal rings) was resected, creating approximately 4-5 mm visual margins cranial and caudal from the gross tumor. The mass and associated trachea were submitted for histopathology. The oral endotracheal tube was exchanged for a sterile 4.5 mm endotracheal tube. The sterile endotracheal tube entering the caudal segment of the trachea through the surgical site was then removed, and the oral endotracheal tube was advanced past the resection site into the caudal segment. The preplaced 4-0 prolene appositional sutures were tightened and were tied off in order to obtain tracheal segment apposition. 4-0 polypropylene sutures were used in a simple interrupted pattern to complete the anastomosis. The dorsal tracheal membrane was friable and did not hold sutures well creating a small tracheal tear. A 1 × 2.5 cm segment of the sternohyoid muscle was harvested and placed over the dorsal aspect of the trachea to reconstruct this defect. This was sutured in place using simple interrupted 4-0 polydioxanone. The endotracheal tube was deflated, repositioned cranial to the tracheal incision, and reinflated. This was used to pressure check the trachea which pressure checked appropriately up to 20 cmH2O. A 12Ga × 20 cm single lumen radiopaque polyurethane thoracostomy tube with multiple fenestrations was placed on the right hemithorax and sutured in place. The surgical site was then routinely closed using 3-0 polydioxanone, 3-0 poliglecaprone, and skin staples. Liposomal bupivacaine was injected along the incisional line during closure. The thoracostomy tube was aspirated until subatmospheric pressure was obtained. Postoperative radiographs were taken and revealed mild tracheal narrowing at the location of the anastomosis (Figure 2).

The cat was extubated and recovered routinely with pulse oximetry (SpO2) readings of 98%. Supplemental oxygen was provided for the first 2 hours after extubation as a precaution. In the oxygen cage, the patient was breathing easily with SpO2 monitoring remaining >98%. The patient was then weaned to room air with SpO2 monitoring remaining >98% overnight with a normal breathing pattern and effort. Postoperative analgesia consisted of a fentanyl CRI for 4hours, a fentanyl transdermal patch (12mcg/h) placed upon recovery, robenacoxib (2 mg/kg SQ), and gabapentin 10 mg/kg PO q8h. Isotonic crystalloids were continued at 1x maintenance (45 ml/kg/24 h) overnight. Cold compressing of the incision continued q4h during his hospital stay. To avoid anxiety-related respiratory complications, the cat was administered trazodone at 5 mg/kg PO q8h postoperatively. This caused excessive sedation, so trazodone was discontinued the following day, and gabapentin was decreased to 5 mg/kg PO q8h. Thoracostomy tube aspirations yielded minimal fluid (<0.25 ml/h) for over 24 h and was removed 24 hours after surgery. Robenacoxib was transitioned to the oral formulation (6 mg PO q24h) the day following surgery. Forty-eight hours after surgery, the cat was alert, eating, and comfortable and was breathing easily with minimal referred upper airway noise. The cat was discharged to the owner on the third day with 3 days of robenacoxib (6 mg PO q24h), 12 mcg fentanyl transdermal patch, 2 weeks of liquid gabapentin (5 mg/kg PO q8-12h), and 2 weeks of liquid amoxicillin/clavulanic acid (13.75 mg/kg PO q12h).

Histopathological analysis of the submitted tissue revealed an incompletely excised squamous cell carcinoma originating from the tracheal mucosa with neoplastic cells noted at the cut edges cranially and caudally. The mass itself is composed of many irregular lobules of neoplastic squamous epithelial cells with central cystic areas within many lobules containing amorphous eosinophilic debris and neutrophils. The neoplastic tissue is noted to have 14 mitotic figures seen per 10 high power fields. This tissue is invading the lamina propria as well as into and through the tracheal cartilage. No lymphatic emboli were noted on the histopathology report. Histopathology of the local cervical lymph node showed no evidence of metastasis. These results were relayed to the owner, and further options including revisional surgery, radiation therapy, or chemotherapy were discussed.

Seventeen days postoperatively the cat was presented for staple removal. The owners stated that the cat was acting normally at home, vocalizing normally and eating/drinking readily. Physical examination revealed a well-healed skin incision. The cat was eupneic with no tracheal sensitivity nor discomfort. No abnormal noise was noted on thoracic and tracheal auscultation. It was recommended to keep the cat's activity restricted for one more week and then reintroduce him back to his normal routine. A consultation with an oncologist was recommended with the option for a revisional surgery vs radiotherapy or chemotherapy being revisited. At the time of last phone recheck (120 days postoperatively), no further treatments have been initiated and a consultation appointment with the oncologist was canceled. The owners were contacted just prior to article submission, and the cat was acting normally at home with no clinical signs of recurrence.

## 3. Discussion

This report describes the first cat with tracheal squamous cell carcinoma to be treated with a tracheal resection and anastomosis and only the third to be treated by any means in the veterinary literature. The procedures described herein resulted in a good short-term outcome for this patient. Feline primary tracheal squamous cell carcinoma is a very rare disease. Including the current report, only 7 cases have been published.  Table 1 summarizes the signalment, treatment, and outcomes of these cases. Due to the paucity of cases, little is known about the prognosis and survival for cats with tracheal SCC; however, some information may be gleaned from these past cases and from the behavior of feline SCC in similar tissues.

^∗^Exact time was not stated in the paper, only that it was euthanized 4-6 weeks after discharge.

Based on histologic tissues of origin, feline oral squamous cell carcinoma may be the most similar acting SCC to tracheal SCC. Feline oral SCC carries a poor prognosis. Local control tends to be the most challenging due to a local aggressiveness and high incidence of bony invasion; however, distant metastasis is rare in cats [32]. There is no known effective treatment that consistently results in adequate control or survival [32]. Chemotherapy appears to have minimal to no effect on feline oral SCC. One study with 5 cats had only 1 partial response and an MST of 30 days when treated with doxorubicin and cyclophosphamide [33]. Eighteen cats treated with a second-generation liposomal cisplatin analog had no response and an MST of 59.8 days [34]. Similarly, radiotherapy alone appears to be ineffective with one study of 9 cats treated with RT having an overall MST of only 86 days [35]. However, other studies do show that when using RT in conjunction with radiation sensitizers, MST can improve slightly up to 112-163 days [36–39].

Surgery, with or without radiation therapy, gains the best results for feline oral squamous cell carcinoma with the extent of resection having major prognostic importance [32]. In 2006, Northrup et al. reviewed 22 cats with oral SCC treated by mandibulectomy alone and showed the median disease-free time after surgery was 340 days [40]. Tumor location and extent of resection had a significant prognostic impact. Rostral tumors had an MST of 911 days, hemimandibulectomy carried an MST of 217 days, which fell to only 192 days when >50% of the mandible was resected. An older study from 1992 by Hutson et al. reviewed cases involving 7 cats with oral SCC treated with a combination of mandibulectomy and RT. In this study, the MST was 14 months (420 days) with a 1-year survival rate of 57% [41]. It was noted that the cause of failure in 86% of these cats was due to local recurrence within 3-36 months (90-1,080 days) after therapy. As such, the general guideline and treatment of choice for feline oral SCC is surgical resection ± radiotherapy.

While chemotherapy and radiation therapy have limited utility in feline oral SCC, neither modality has been attempted in feline tracheal SCC and, as such, no data on their efficacy is available. The treatment of choice for human tracheal SCC, however, is surgery and/or radiation therapy, lending support of these therapies for feline patients [42, 43]. In one human study that combines SCC and adenoid cystic carcinomas, 16% of patients had surgery alone, 42% had surgery combined with radiation therapy, 23% had radiation therapy alone, and 18% had unknown treatments [43]. In that cohort, the 5-year survival rate was 40% with a median survival time of 30 months. In a more recent study, the 5-year survival rate was 50% for patients who had surgery alone (*n* = 6), 50% for surgery combined with RT (*n* = 3), and 0% for RT alone (*n* = 2). This hints that surgery may be the more effective primary treatment with RT being relegated to an adjunctive therapy only [42]. This fits well with the presumptive information based on feline oral SCC.

Tracheal stenting has been reported for 2 feline tracheal masses; an adenocarcinoma by Scherer et al. [27] and a carcinoma by Culp et al. [30]. Both were palliative only measures, and the patients did recover well from both procedures, going back to normal respiratory effort. The cat with the adenocarcinoma had the primary mass debulked endoscopically 3 times over the first 6 months, and then, the stent was placed. The stent allowed for 6 additional months of comfort and normal breathing prior to the recurrence of clinical signs. A fourth debulking procedure was attempted, but the patient died during the procedure. Necropsy showed the tracheal mass as well as pulmonary metastasis [27]. The carcinoma case had the stent placed at the time of diagnosis. The patient was normal for 6 weeks until a sudden onset dyspnea occurred. Pulmonary metastasis was suspected as the cause; however, the patient was euthanized at this time with no necropsy to confirm this suspicion [30]. These cases show that intraluminal tracheal stenting can be performed with good short-term results, returning the patients to clinically normal for some period of time. The procedure is fast and minimally invasive; however, the time of palliation may be highly variable. Another consideration to take into account when discussing a stent placement with the owners is that once an intraluminal stent is placed, future tracheal R&A surgery is no longer an option due to the ingrowth of the trachea into the stent. As such, intraluminal stenting should be reserved for end of life palliation or salvage procedures.

Based on the 2 reported cases of feline tracheal SCC palliated with endotracheal debulking [4, 8], this modality can have good short-term resolution of clinical signs (Table 1). The advantages of endoscopic snare debulking include speed and decreased morbidity while allowing for a therapeutic option that can also gain a biopsy sample to definitively diagnose the mass prior to a potential surgery. The procedure carries few risks with the most common being a mild, self-resolving hemorrhage and the most severe being the possibility for a tracheal tear. The main disadvantage of this therapy is the short-term results with the mass regrowing quickly (28-60 days) [4, 8]. Additionally, consecutive debulking procedures may observe the law of diminishing returns based on the single case by Howard et al. [4]. A complete histopathological characterization describing the differentiation of the tumors in these two cats is not available. Unfortunately, this means that a direct comparison of the disease-free interval (DFI) and survival time between cats treated with debulking versus R&A cannot be made. It is of note, however, that the cat treated with surgical R&A did achieve a DFI more than twice as long as either of the 2 cats treated with endoscopic debulking (Table 1). Hence, based on the limited information available, aggressive surgical management by tracheal resection and anastomosis with wide margins ± RT may be the only way to achieve any long-term disease-free interval, let alone a cure.

Of the 56 reported feline tracheal masses, 11 have been treated with tracheal R&A. Of these, 6 cats, including the one described in this case report, were normal at their last follow-up (range 3-32 months) [4, 18, 19, 25, 29]. One cat with lymphoma had no additional treatment and was euthanized 4 months later due to lymphoma dissemination to other internal organs [21]. On necropsy, this cat had no detectable neoplasia in the trachea. Two cats with incomplete margins had regrowth of their tracheal mass at 3 and 12 months after surgery [8]. The remaining two cats had severe postoperative complications (cardiac arrest twice postoperatively in one and iatrogenic laryngeal paralysis in the other) and were ultimately euthanized 4 and 13 days postoperatively, respectively [8, 20]. This data indicates that most cats that survive to discharge can have good long-term survival with 7/9 (77.8%) having no clinical signs of tracheal disease at the time of last follow-up (range 3-32 months) (Table 2).

While surgical excision of a tracheal SCC with a tracheal R&A has only been described in the present case, it does appear that wider surgical margins are necessary with this tumor type and location. In the current case, 5 tracheal rings (~3 cm) were resected, removing the gross lesion with 4-5 mm margins cranially and caudally. On histological review, the margins were incomplete with neoplastic cells advancing to the edge of the excised tissue both cranially and caudally. Incomplete margins have been the trend in the past and current literature for feline tracheal masses in general. Of the 11 cases of feline tracheal masses treated with surgical R&A, one case of a basal cell carcinoma with a 4 ring resection reports a “marginal” resection [29], 5 cases with 3 to 7 ring resections report incomplete margins [8, 19, 25, 29, 31], and the last 5 cases with 4 to 10 ring resections do not comment on the margins [4, 8, 18, 20, 21] (Table 2).

While the length of the trachea that can be safely resected in a domestic cat has not been well studied, there have been case reports stating good outcome when removing up to 7-8 tracheal rings [8, 19]. There are canine studies which show that it can be safe to remove up to 50% of the trachea in the dog with only minimally affecting the intraluminal diameter; however, the position of the lobar bronchi changes on endoscopic examination once 30% or more of the trachea has been removed [44]. De Mello-Souza and Reinero postulate that the annular ligaments are able to stretch under tension and can compensate for an approximately 20% tracheal resection, but anything greater appears to pull the lungs cranially, displacing the lobar bronchi [44]. The clinical significance of this cranial displacement has not been confirmed to the author's knowledge. Studies in sheep also report a similar outcome as in dogs, being able to remove as much as 50-58% of the adult tracheal length without increased postsurgical complications [45]. While these studies do not necessarily correlate to feline tracheas, a conservative tracheal resection of up to 30% has been suggested for cats [4, 46]. Given that cats typically have 38-42 tracheal rings [47], the conservative estimate would then allow for up to 11-13 rings to be resected. Using this guideline and reviewing Table 2, at least 9 of the cases could potentially have had more tracheal rings removed which may have allowed margins to be reached. It is worth noting, however, that the one case with 10 tracheal rings resected developed iatrogenic, bilateral laryngeal paralysis. This highlights the need for meticulous surgical technique, especially if larger resections are attempted. As a way to improve healing in cases of larger resections, the use of tension-relieving sutures may be used to decrease the tension at the anastomosis site, reducing the potential for dehiscence [46].

## 4. Conclusion

Feline tracheal squamous cell carcinoma is a very rare neoplasm with only 7 cases now reported. This report describes the first cat with tracheal SCC in veterinary literature to be treated with a tracheal resection and anastomosis and only the third case to have any form of treatment. Tracheal R&A for feline tracheal SCC appears to require margins greater than 5 mm based on the current report alone. Even with incomplete margins, however, tracheal R&A may prolong clinical remission/disease-free interval when compared to endoscopic debulking alone. It is hypothesized by the authors that larger resections could allow for complete margins which may, in turn, further improve the clinical remission/disease-free interval. Further cases with longer follow-up will be needed to support and confirm this hypothesis.

## Figures and Tables

**Table 1:** Primary feline tracheal squamous cell carcinoma.

Source	Signalment	Treatment and outcome	Survival
Veith et al. 1974	2YO DSH	N/A	0 days
Lobetti et al. 1992	N/A	Euthanized with no treatment	0 days
Jakubiak et al. 2005	11.5YO FS DSH	Euthanized with no treatment	~7 days
Jelinek et al. 2012	11YO FS DSH	Euthanized with no treatment	0 days
Queen et al. 2010	16YO MC DSH	Debulked with an endoscopic snare. Clinical remission for 4 weeks until SCC regrowth and severe dyspnea recurrence.	28-42 days^∗^ postsurgery
Howard et al. 2017	9YO MC Bengal	No treatment for a month. Endoscopic debulking led to clinical remission for 2 months. Another endoscopic debulking allowed for an additional 20 days of clinical remission until severe dyspnea recurred and the patient was euthanized.	CR for 60 days after initial debulk, CR 20 days after second debulk (110 days total)
Current report	10YO MC DSH	Tracheal resection and anastomosis. Margins incomplete. Referred to oncology to discuss further options including revisional surgery, radiation therapy, and chemotherapy. No further treatment had been initiated at the time of last phone recheck (120 days postoperatively)	In CR at the time of last phone update (120 days postop)

Abbreviations: CR: clinical remission: DSH: domestic shorthair; FS: female spayed; MC: male castrated; N/A: not available; YO: year old.

**Table 2:** Outcomes for feline tracheal masses treated with tracheal R&A.

Source	Diagnosis	# of rings or cm resected	Margins on histopathology	Additional therapy	Outcome
Schneider 1979	Histiocytic LSA	N/A	N/A	Chemo-therapy	Clinical remission at 8 months
Beaumont 1982	Lymphoblastic LSA	1.8 cm	N/A (palpable mass was 1.5 cm)	None	Systemic LSA, euthanized 4 months later. On necropsy, no neoplastic cells in the trachea.
Katayama et al. 2013	B cell LSA	10 rings	N/A	None	Iatrogenic bilateral laryngeal paralysis. Patient euthanized 13 days after surgery
Bataller et al. 2017	B cell LSA	7 rings	Incomplete	Chemo-therapy	CR at 20 months
Howard et al. 2017	LSA	4 rings	N/A	None	In CR at discharge then lost to follow-up.
Evers et al. 1994	ACA	3 rings	Incomplete	None	CR at 3 months before lost to follow-up
Queen et al. 2010	ACA	8 rings	Incomplete	Debulked with snare after regrowth	CR for 12 months before regrowth. Endoscopic snare debulking was performed. Euthanized 35 months after the initial diagnosis.
Queen et al. 2010	ACA	N/A	N/A	None	Euthanized 4 days postoperatively due to cardiac arrest twice postop.
Essman et al. 2002	Leiomyo-sarcoma	3 rings	Incomplete	None	CR for 3 months before dyspnea. Radiographs showed regrowth and the cat was euthanized.
Green et al. 2012	Basal cell carcinoma	4 rings	“Marginal”	None	CR 32 months postop.
Current report (Miller et al. 2020)	Squamous cell carcinoma	5 rings	Incomplete	None	CR 4 months postop.

Abbreviations: ACA: adenocarcinoma; CR: clinical remission; LSA: lymphosarcoma; N/A: not available.
